# Comprehensive bioinformatics analysis of malignant transformation and potential therapeutic possibility of lung adenocarcinoma after lipopolysaccharide induction

**DOI:** 10.3389/fgene.2025.1556366

**Published:** 2025-07-08

**Authors:** Wanjie Xu, Jing Zhang, Xinyu Zhang, Xue Wang, Yiluo Xie, Shengping Min, Xiaojing Wang, Chaoqun Lian

**Affiliations:** ^1^ Department of Clinical Medicine, Bengbu Medical University, Bengbu, China; ^2^Anhui Province Key Laboratory of Clinical and Preclinical Research in Respiratory Disease, The Department of Pulmonary Critical Care Medicine, First Affiliated Hospital of Bengbu Medical University, Bengbu, China; ^3^ Department of Genetics, School of Life Sciences, Bengbu Medical University, Bengbu, China; ^4^ Research Center of Clinical Laboratory Science, Bengbu Medical University, Bengbu, China; ^5^ Digestive Department, Xi’an Fifth Hospital, Xi’an, China; ^6^ Molecular Diagnosis Center, Joint Research Center for Regional Diseases of IHM, The First Affiliated Hospital of Bengbu Medical University, Bengbu, China

**Keywords:** lung adenocarcinoma, bioinformatics, lipopolysaccharide, trans-differentiation therapy, adipogenic, epithelial-mesenchymal transition

## Abstract

**Background:**

Lipopolysaccharides are involved in malignant progression and epithelial-mesenchymal transition of cancer. The mechanism of LPS in malignant progression of lung adenocarcinoma and possible therapeutic strategies need to be explored.

**Methods:**

After obtaining LPS-induced characteristics, 850 samples were characterized. Differences in features were evaluated in different risk subgroups. Cell lines with high scoring performance were selected for *in vitro* experimental validation and possible potential therapeutic options were identified.

**Results:**

By using bioinformatics analysis to obtain LPS signature genes from the LPS induction cohort, the five intersecting genes were utilized to construct a risk model associated with LPS induction. The high-risk score subgroup had a poorer prognosis and lower immunotherapy response, and this subgroup showed distinct EMT features such as hypoxia pathway, high enrichment of WNT pathway and high TP53 mutation. After verifying that the risk model has a close correlation with EMT progression, we obtained cell lines with high EMT-associated features, confirming the possibility of LPS-induced EMT, i.e., demonstrating that LPS acts in inducing EMT progression. The malignant progression of tumors could be inhibited using rosiglitazone and liraglutide combined with lipid-forming trans-differentiation therapy.

**Conclusion:**

In the field of bioinformatics, our study acquired genes characteristic of lipopolysaccharide-induced lung adenocarcinomas and elucidated for the first time that LPS-induced associated scoring patterns correlate with EMT progression. In addition, we propose the possibility of treatment with trans-differentiation therapies that utilize the high plasticity that EMT progressing cancer cells have.

## Introduction

Lung cancer is a disease that has a wide range of clinicopathological features and is one of the highest morbidity and mortality rates. Non-small cell lung cancer (NSCLC) is the most prevalent form of lung cancer globally, making up around 55%–60% of all lung cancer fatalities. Lung adenocarcinoma (LUAD) is a type of NSCLC that has unique morphological features, metastatic patterns and clinical outcomes, and its survival rate remains low (Denisenko et al., 2018). When lung adenocarcinoma is identified, it is common for patients to be in the metastatic phase, with metastases to the brain, bones, and respiratory system that are distant (Riihimäki et al., 2014), at which point cancer can no longer be treated by surgically removing the malignant tumor (Osmani et al., 2018; Li et al., 2022). Consequently, there is a need for further investigation into molecular markers and strategies for lung adenocarcinoma.

Tumor metastasis occurs due to dynamic changes, which are achieved through a combination of cancer cell signaling cascade responses and the tumor microenvironment. Epithelial-mesenchymal transition (EMT) is a dynamic process in which cells gradually lose their epithelial characteristics and exhibit mesenchymal properties. This process plays an important role in promoting cancer cell plasticity and, under certain conditions, can promote distant metastasis and escape chemotherapy by inducing the process of de-differentiation and signaling adaptation in cancer cells (Puram et al., 2017; Ishay-Ronen et al., 2019). Furthermore, studies have demonstrated that the tumor microenvironment associated with inflammation fosters aggressiveness in various forms of cancer. Non-small cell lung cancer patients frequently acquire bacterial pneumonia, leading to a decline in treatment effectiveness and prognosis. Research has demonstrated that individuals with NSCLC who have not been infected with bacterial pneumonia have a survival rate of more than 30% after 28 months of treatment. Still, those infected with pneumonia have a survival rate of only 10% (Chen et al., 2021). As a major bacterial pathogen, lipopolysaccharide (LPS) is a component of the cell membrane of Gram-negative bacteria that drives nuclear factor (NF)-κB and induces the production and release of a variety of pro-inflammatory mediators, e.g., tumor necrosis factor (TNF)-α, interleukin (IL)-6 and IL-1 (Pchejetski et al., 2011; Vlachostergios et al., 2013). Research has shown that the presence of inflammation in the tumor microenvironment caused by LPS can lead to the development of various epithelial cancer types and increase the invasiveness of cancer cells, including pancreatic and gastric cancers (Pchejetski et al., 2011; Liu et al., 2017; Li et al., 2019). Meanwhile, LPS has been shown to increase tumor invasiveness by inducing the EMT transition in many cancers (Chen et al., 2012; Jing et al., 2012; Chen et al., 2021; Peng et al., 2023), but its role in lung adenocarcinoma and the underlying mechanisms have not yet been clearly depicted. Recently, Nemanja Despot Marjanovic et al. found that during lung cancer development, a subset of cancer cells with EMT progression had a highly mixed state and high plasticity of production (Marjanovic et al., 2020). Overall, the phenotypic transformation of EMT-progressing lung adenocarcinoma cells is a critical step in their invasiveness and drug resistance (Shaurova et al., 2020; Xiao et al., 2023), and the phenotypic transformation of cancer cells is usually accompanied by the generation of high plasticity. It remains to be explored whether LPS leads to phenotypic transformation of lung adenocarcinoma, as well as the underlying molecular mechanisms and the outcome of high plasticity.

Differentiation therapy means that hormones or cytokines may promote differentiation *in vitro*, thereby irreversibly altering the phenotype of the cancer cells, e.g., the combination of retinoic acid and arsenic, which can be highly curative of acute promyelocytic leukaemia, is a hallmark success of differentiation therapy (de Thé, 2018). The use of high plasticity in the EMT state to transform highly invasive cancer cells into less invasive ones has become a novel approach to cancer treatment, given that tumors frequently experience EMT during malignant progression. Considering that LPS induces highly invasive cancer cells and its association with EMT in lung adenocarcinoma has not yet been clarified, a comprehensive description of the TME environmental adaptations, molecular dynamics of crossover, and exploration of potential therapeutic options in the context of LPS-induced malignant transformation of lung adenocarcinomas would be helpful in exploring therapeutic strategies for lung adenocarcinomas.

In this study, we used the LPS induction cohort to obtain the characteristic genes for LPS induction in lung adenocarcinoma. Based on the five cross-cutting LPS-induction associated genes which are differentially expressed in lung adenocarcinoma and significantly affect the prognosis of lung adenocarcinoma, we developed an innovative scoring system to measure the progression of tumors in five distinct cohorts, while also identifying distinct expression patterns among subgroups in clinical characteristics, molecular processes, TME infiltration landscapes, gene mutations, and immunotherapy predictions to direct patient treatment plans. At the *in vitro* experimental level, we successfully constructed a model of highly invasive cancer cells in the context of EMT on the basis of the LPS risk model and suggested the possibility of a novel therapy. In conclusion, they will enhance our comprehension of the role and probable processes of LPS in the malignant transformation of lung adenocarcinoma and offer novel concepts for the formation of more efficient treatment approaches.

## Materials and methods

### Data collection and pre-processing

Lung adenocarcinoma sample sequencing data and clinical characterisation were collected through publicly available datasets from the NCBI GEO (https://www.ncbi.nlm.nih.gov/geo/), TCGA (https://cancergenome.nih.gov/) databases. The TCGA cohort, GSE50081 cohort (Der et al., 2014), GSE31210 cohort (Okayama et al., 2012), IMvigor210 cohort (Mariathasan et al., 2018), GSE78220 cohort (Hugo et al., 2016), and GSE132661 cohort RNA data were processed as separate samples. Lung adenocarcinoma RNA sequencing data from the TCGA cohort were collected through The Genomic Data Commons (https://portal.gdc.cancer.gov/) in The Cancer Genome Atlas (TCGA), and subsequent analyses were performed using the TPM format of the RNA sequencing data. RNA sequencing data for lung adenocarcinoma samples, LPS-treated lung cancer samples, and immunotherapy samples were collected from the GEO databases GSE50081, GSE31210, GSE132661, and GSE78220. Transcripts Per Million (TPM) values for the transcriptomes of the LUAD cell lines were obtained from the Cancer Cell Line Encyclopedia (CCLE) (https://sites.broadinstitute.org/ccle/). Analyses were performed using the TCGA lung adenocarcinoma cohort as well as the GEO lung adenocarcinoma cohort, and both the IMvigor210 cohort and the GSE78220 cohort were used in the immunotherapy module.

### WGCNA and candidate hub genes identification

Co-expressed gene networks in the GSE132661 cohort representing the control, lung cancer, control lung cancer and LPS-treated lung cancer groups were identified using the WGCNA R package (v1.72-1) (Langfelder and Horvath, 2008). The top 20,000 genes with mean absolute deviation (MAD) were screened for network construction and analysis. Genes from the green module were selected as LPS-induced hub genes. Gene Ontology (GO) enrichment analysis was performed on the hub genes in the co-expression module using the clusterProfiler R package (v4.6.2) (Yu et al., 2012).

### Differential expression analysis and screening of signature genes

To identify Differentially Expressed Genes (DEGs) in lung adenocarcinoma, the DESeq2 package (v1.36.0) was utilised to assess differentially expressed genes between subgroups. The significance screening criteria for related genes were adj.P.Value less than 0.05 and |logFC|>1.5. The LPS-induced hub genes were transformed using the homologene R package (v1.4.68). Hub genes were compared with differential genes and intersections were obtained. Hub genes associated with overall survival (OS) were further screened using the Univariate Cox regression analysis in TCGA cohort and GSE50081 cohort (P < 0.01) and defined as LPS induction-related signature genes.

### Construction and validation of the LPS-induction model

The Least absolute shrinkage and selection operator (LASSO) cox regression analyses were performed using the R packages glmnet (v4.1-8), survival (v3.5-7), and survminer (v0.4.9), which were used to construct a risk model associated with lipopolysaccharide induction in lung adenocarcinoma. We derived the risk score formula through analysis: Risk Score = Ʃ (βi × Expi), where βi denotes the regression-weighted coefficient of each prognostic marker, and Expi corresponds to its expression level. The prognostic value of the score was validated in the TCGA cohort, GSE50081 cohort, GSE31210 cohort, IMvigor210 cohort, and GSE78220 cohort. The division into high and low risk groups is based on the median value of the risk score.

For genes in the LPS-induction model, Kaplan-Meier analyses were performed using the survival (v3.5-7) package to assess the association with overall survival (OS) in terms of median high and low mRNA expression groups. The diagnostic value of genes was assessed using the Receiver Operating Characteristic Curve (ROC) analysis using the pROC (v1.18.4) R package. In addition, the results of immunohistochemical analysis of PRC1 protein expression in normal versus LUAD tissues were downloaded from The Human Protein Atlas (HPA) database (http://www.proteinatlas.org/).

### Differences in subgroup molecular pathways

Well-defined biosignatures were derived from the Hallmark gene set (h.all.v2023.1.Hs.symbols). EMT-related gene sets were obtained through the Molecular Signatures Database (MSigDB, http://software.broadinstitute.org/gsea/msigdb/), including ANASTASSIOU_MULTICANCER_ INVASIVENESS_SIGNATURE(Anastassiou et al., 2011), FOROUTAN_INTEGRATED_TGFB_EMT_UP(Foroutan et al., 2017), FOROUTAN_PRODRANK_TGFB_EMT_UP(Foroutan et al., 2017), FOROUTAN_TGFB_EMT_UP(Foroutan et al., 2017), LEF1_UP.V1_UP, SARRIO_ EPITHELIAL_MESENCHYMAL_TRANSITION_UP(Sarrió et al., 2008). The GSVA (v1.44.5) R package was utilised to investigate changes in biological processes between different groups. PROGENy enrichment was performed using the progeny (v1.18.0) package to quantify signalling pathway target gene enrichment to further clarify pathway alterations between subgroups (Schubert et al., 2018). The Gene set enrichment analysis (GSEA) was performed using limma (v3.54.0), and clusterProfiler (v4.6.2) R packages to further explore the potential association of LPS-induced models with EMT. Correlation analyses of risk scores, model gene and pathway enrichment scores were performed using the ggplot2 (v3.4.3), and ggpubr (v0.6.0) R packages.

### Tumor microenvironment (TME) infiltrations exploration

The ESTIMATE (v1.0.13), IOBR (v0.99.9) R package was used to perform ESTIMATE algorithms and Immuno-Oncology Biological Research (IOBR) analyses in order to investigate tumor microenvironmental characteristics of each LUAD sample. The IOBR package (Zeng et al., 2021) integrates eight published algorithms for quantifying the tumor Microenvironment (TME) algorithms: CIBERSORT, TIMER, xCell, MCPcounter, ESTIMATE, EPIC, IPS, quanTIseq for a more comprehensive analysis of cellular infiltration levels in TME.

### Somatic mutation analysis

Somatic mutation and CNV data of the TCGA cohort were downloaded from GDC TCGA (https://cancergenome.nih.gov/), and the data were acquired to explore the variability of mutations between subgroups using the maftools (v2.12.0) R package. The cancer-immunity cycle was obtained from Xu et al., and scores were obtained by expression profiling for inter-subgroup comparisons.

### Cell culture and induction programme

*In vitro* culture experiments were performed using A549 cells cultured in DMEM medium and RPMI 1640 medium (Gibco, ThermoFisher Scientific, United States) supplemented with 10% foetal bovine serum, 1% penicillin and streptomycin (Gibco). Logarithmic growth phase A549 cells were collected and inoculated in 6-well cell culture plates and placed in an incubator.

When the cell density was appropriate, the old medium was discarded, and medium containing 10 μg/mL and 20 μg/mL LPS was added sequentially. And medium without LPS was used as a control and placed in an incubator for 48 h and 72 h, respectively; cancer cells inducing the epithelial-mesenchymal model were obtained, and the medium containing 10 μmol/mL and 20 μmol/mL rosiglitazone and liraglutide were added, and the DMEM medium was used as a control, and placed in an incubator for 48 h, 72 h and 96 h, respectively.

### Western blot and RT-qPCR

RNA was extracted from the lung adenocarcinoma cell line (A549) as a control. The cDNA was synthesised for real-time PCR using SYBR Green qPCR mix (Vazyme, China). cDNA was primed as follows: UCP1-Forward: AGG​TCC​AAG​GTG​AAT​GCC​C; UCP1-Reverse: TTA​CCA​CAG​CGG​TGA​TTG​TTC; C/EBPα- Forward: AAA​CAA​CGC​AAC​GTG​GAG​A; C/EBPα-Reverse: GCG​GTC​ATT​GTC​ACT​GGT​C; FABP4-Forward: ACT​GGG​CCA​GGA​ATT​TGA​CG; FABP4-Reverse. CTC​GTG​GAA​GTG​ACG​CCT​T; GAPDH-Forward: GAC​CAC​AGT​CCA​TGC​CAT​CA; GAPDH-Reverse: GTC​AAA​GGT​GGA​GGA​GTG​GG. Protein blotting analyses were performed on RIPA cleavage buffer (Servicebio, China) containing PMSF (Servicebio) buffer (Servicebio, China) was used to collect proteins from A549 cells. 10% sodium dodecyl sulphate-polyacrylamide gel electrophoresis (SDS-PAGE) was used to separate the protein samples, and polyvinylidene difluoride membrane (PVDF) membranes (Immobilon-P, Carlsbad, Ireland) were used to transfer the separated proteins. The membranes were blocked for 15 min using a rapid blocking solution and then incubated with primary antibodies: E-Cadherin (1:1,000), N-Cadherin (1:1,000), Vimentin (1:1,000) overnight at 4°C, followed by 2 h of incubation with secondary antibodies.

### Transwell migration invasion and wound healing assay

Transwell migration and wound healing assays A549 cells were performed after 72 h of LPS induction, 72 h of Rosi versus Lira induction, and cultured in 24-well culture plates with 8 mm pore membrane inserts to measure cell migration and invasive capacity. 4 x 10^4 cells were inoculated in the upper chamber of a transwell with 200 ul of serum-free medium, and 800 μL of medium containing 10% FBS was added to the lower chamber. After 48 h of incubation, cells migrating across the membrane were fixed with paraformaldehyde, stained with 1% crystal violet and counted under a light microscope (200×). In addition, A549 cells were cultured in 24-well plates and scraped with a 200 ul pipette tip. Cells were cultured in DMEM and RPMI 1640 medium without FBS. Wound images were captured at 0 and 24 h, and the wound area was quantified by ImageJ software (40×).

### CCK-8 experiment

The induced A549 cells were planted in 96-well plates at 4 × 103/well, six replicate wells were designed, and four groups were repeated, and 10 μL of CCK-8 reagent was added to each well of the four groups after 4 h, 24 h, 48 h, and 72 h, respectively, and placed in the incubator for 2 h, and at the end of the incubation, the absorbance value was measured by using an enzyme labeller set to 450 nm, and the results of the experiments were recorded.

### Statistical analyses

Statistical analyses and academic graphing were performed in R software (v3.6.3) and GraphPad Prism 8.0. Two-way comparisons between the two groups were performed using the Wilcoxon test, and t. test, and survival analyses were performed using Kaplan-Meier method and log-rank test. Statistical significance of cell line experiments was assessed by GraphPad Prism version 9 software. Differences were considered statistically significant at *p < 0.05, **p < 0.01, ***p < 0.001, ****p < 0.0001.

## Results

### WGCNA identifies signature genes

**FIGURE 1:**
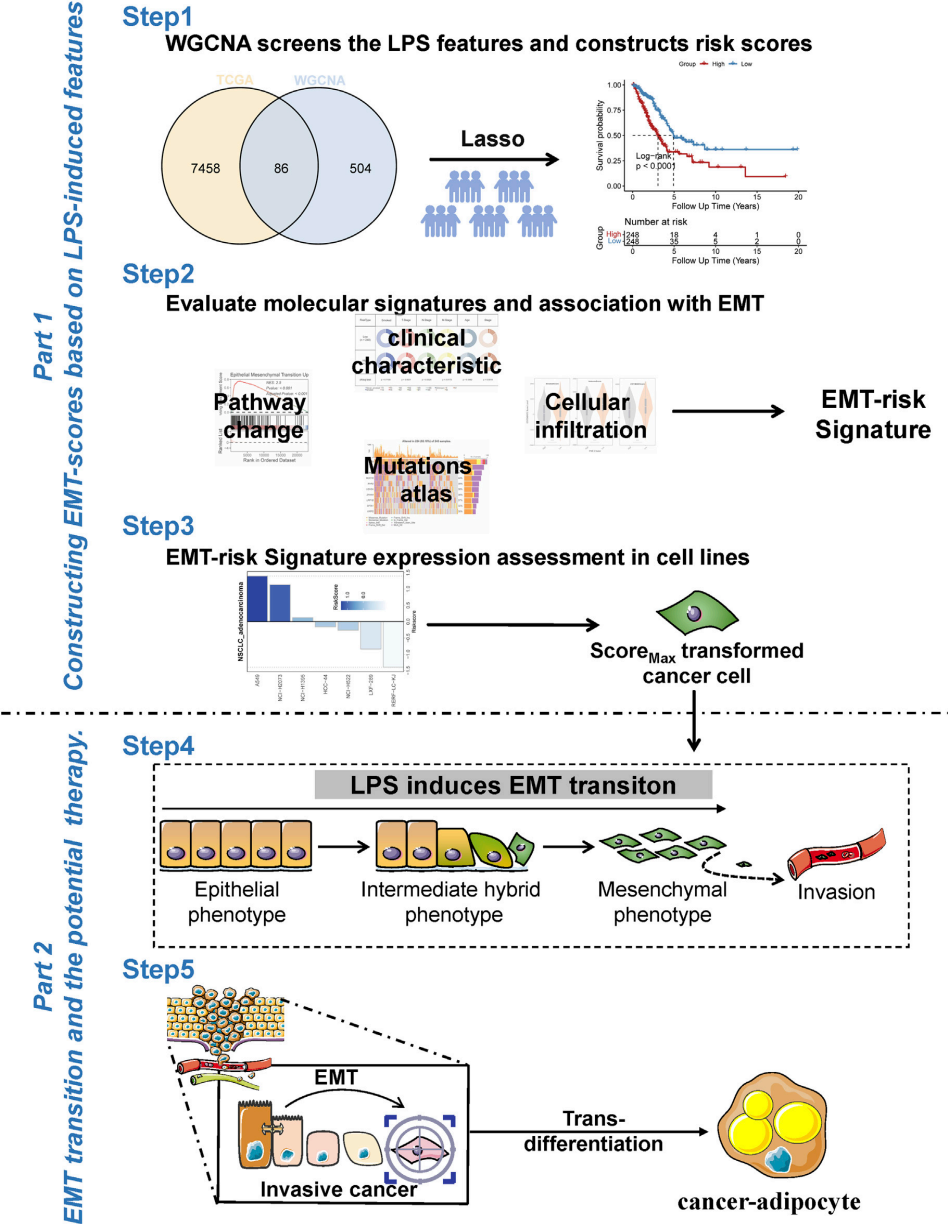
Work flow of this study. Overall workflow diagram of the study for better understanding of the process. Portions of the figure utilized images from Servier Medical Art, licensed under CC BY 4.0.

**FIGURE 2:**
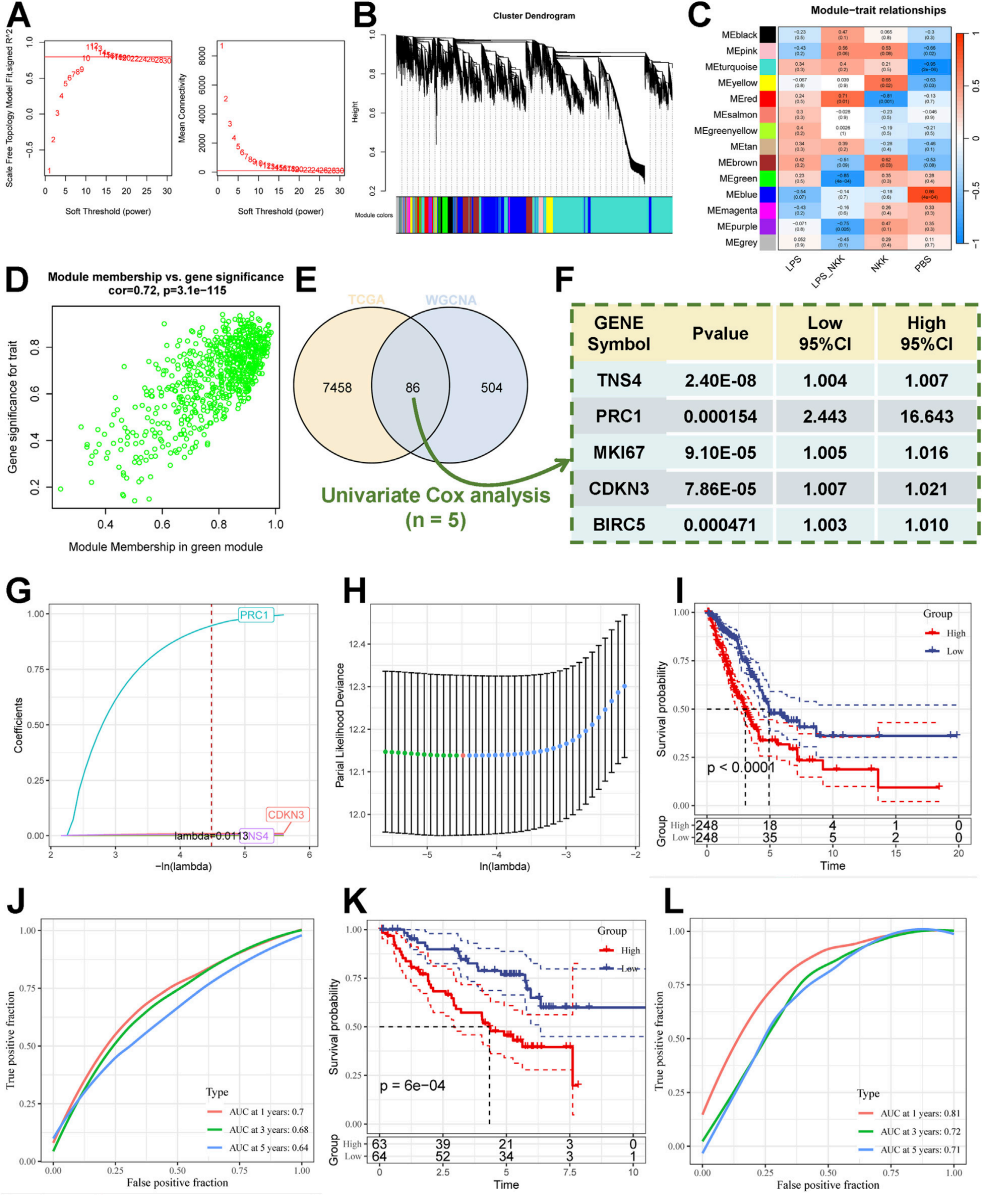
Modeling the risk associated with LPS induction. **(A)** Scale independence and mean connectivity of multiple soft-thresholding powers (β) from 1 to 30. **(B)** The cluster dendrogram developed by the weighted correlation coefficients, genes with similar expression patterns were clustered into co-expression modules, and each color represents a module. **(C)** Heatmap of the correlation between module eigengenes (MEs) and Experimental group. **(D)** Scatter plot displaying relationship of module membership (MM) in green module with gene significance. **(E)** Venn diagram of DEGs of LUAD with green module genes. **(F)** Results of Univariate Cox regression analysis of characterised genes. **(G,H)** LASSO algorithm to derive 3 model genes. **(I)** Kaplan-Meier curves for the high and low-risk score groups in the TCGA cohort. **(J)** the Receiver Operating Characteristic Curve analysis for TCGA cohorts. **(K)** Kaplan-Meier curves for the high and low-risk score groups in the GSE50081 cohort. **(L)** the Receiver Operating Characteristic Curve Analysis for GSE50081 cohorts.

Prior to the commencement of the entire study, we summarised the design ideas of the study as well as the overall workflow in order to provide an overview to explore the potential impact of LPS-induced features on lung adenocarcinoma as well as the potential therapeutic potential (Figure 1). We obtained and analyzed data from the GSE132661 cohort, which consists of a mouse model of LPS-induced lung cancer that was induced with 4-(methylnitrosamino)-1-(3- pyridyl)-1-butanone (NNK) (the PBS-treated group served as a control group), and using LPS to induce the development of chronic inflammation. Subsequently, WGCNA was employed to ascertain genes that could be identified as LPS-inducible within the GSE132661 cohort. Firstly, the optimal soft threshold power β was set to 11 to ensure the scale-free network constructions (Figures 2A,B; Supplementary Figure S1A). The clustering dendrogram shows that genes with similar expression patterns were clustered into 15 modules. Among the 15 modules, the green module had the strongest correlation with the LPS-induced lung cancer group (NKK_LPS) in absolute value (ME = −0.85, p = 4e-04) (Figure 2C), while the green module had the strongest correlation with gene signatures (cor = 0.72, p = 3.1e-115) (Figure 2D). Consequently, the green module is chosen as the central module and is further examined.

To explore the biological processes of the green module hub genes, we performed GO pathway enrichment analysis. Biological processes (BP) major enrichment results showed that hub genes were mainly associated with cell growth and motility (Supplementary Figure S1B), including negative regulation of growth, negative regulation of developmental growth, regulation of ion transmembrane transporter activity, etc. The results indicate that green module hub genes are associated with cell growth and motility, which is consistent with our goal to screen for genes associated with LPS-induced cancer cell progression from a mouse model of LPS-induced lung cancer.

We further analysed the Differentially Expressed Genes (DEGs) of LUAD samples versus normal samples in the TCGA cohort (threshold |logFC| > 1.5, adj.P.value <0.05), and obtained a total of 7,544 DEGs. DEGs were compared with hub genes, and intersections were obtained (Figure 2E; Supplementary Table S1A–C) to further screen the hub genes associated with lung adenocarcinoma. Meanwhile, we further screened the genes associated with overall survival (OS) using univariate Cox regression analysis. The intersections of LPS induction-related genes, lung adenocarcinoma differential genes, and univariate cox genes were obtained, and five genes (TNS4, PRC1, MKI67, CDKN3, BIRC5) were obtained, which were defined as the characteristic genes associated with LPS induction (Figure 2F). Based on the LPS-induced signature genes to enable subsequent risk modeling.

### Construction and validation of the LPS-induction model

We employed the Least absolute shrinkage and selection operator (LASSO) Cox regression analysis for the TCGA cohort to construct the LPS-induction model using the five signature genes as input genes. The optimal lambda value was determined automatically with minimum bias by 10-fold cross-validation, and 2 genes with weak survival signals were excluded. The final output model was: Riskscore=(1.031)*exp (PRC1)+(0.01)*exp (CDKN3)+(0.005)*exp (TNS4) (Figures 2G,H). The median score was used to calculate the score of each lung adenocarcinoma patient, which was then divided into high-risk and low-risk subgroups (Supplementary Table S2A–C). Kaplan-Meier analyses were conducted to investigate the prognostic variability of the high- and low-risk subgroups, and the Receiver Operating Characteristic Curve (ROC) was used to evaluate the model’s predictive capability. The results showed that the prognosis of the high-risk subgroup in the TCGA cohort (Figure 2I) was significantly worse than that of the low-risk subgroup (log-rank test, P < 0.0001). We validated the OS prediction ability of the LPS-induction model in other cohorts, and the OS time of patients in the high-scoring subgroups in the GSE50081 cohort (Figure 2K) (log-rank test, P = 6e-4) and the GSE31210 cohort (Supplementary Figure S1C) (log-rank test, P = 0.0019) was significantly lower than that in the low-risk subgroup. Meanwhile, the model had good 1-year, 3-year, and 5-year in TCGA cohort (Figure 2J) (ACU = 0.7, 0.68, 0.64), GSE50081 cohort (Figure 2L) (AUC = 0.81, 0.72, 0.71), and GSE31210 cohort (Supplementary Figure S1D) (AUC = 0.70,0.68,0.75) predictive value.

**FIGURE 3:**
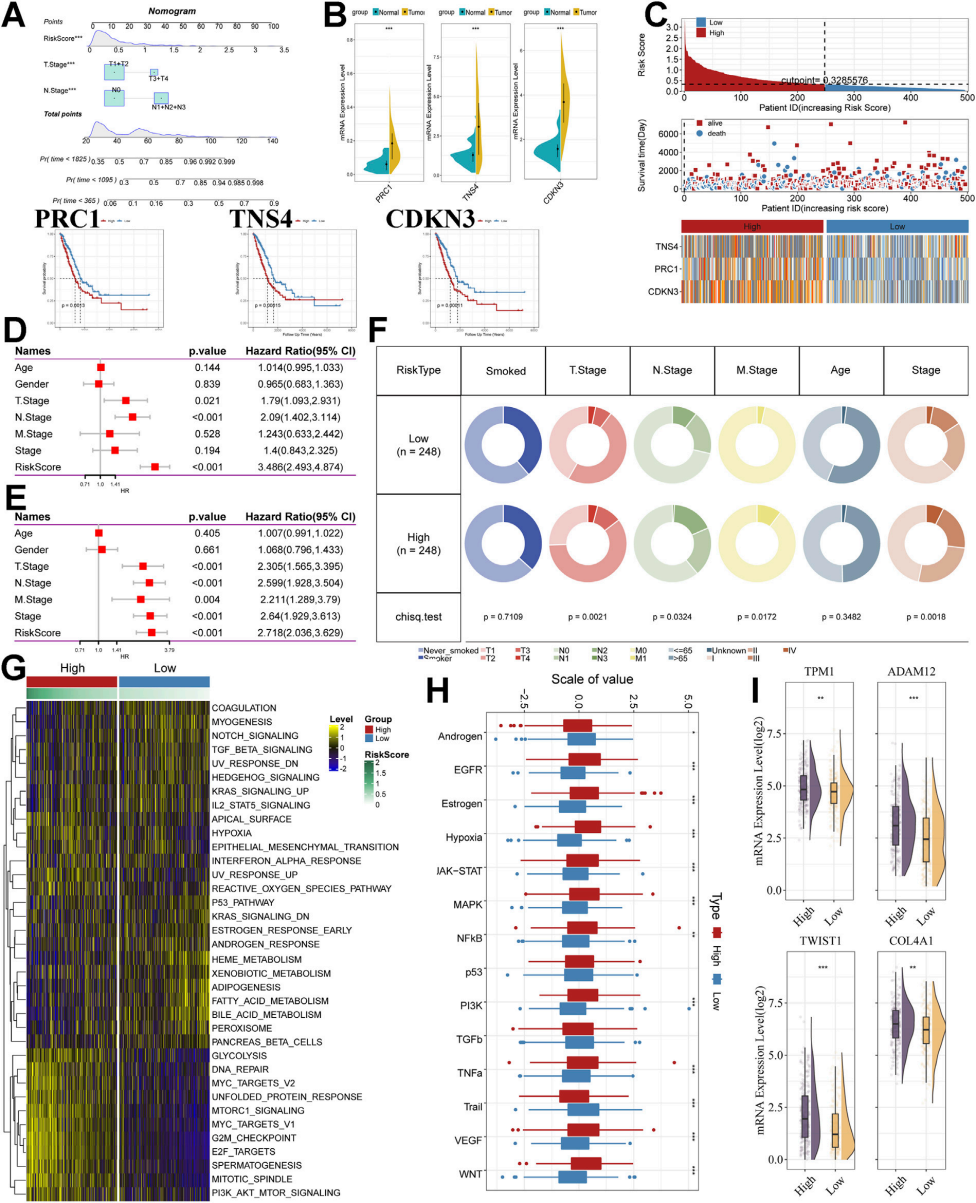
Analysis of model stability, clinical, and molecular alterations. **(A)** Nomogram of Riskscore versus clinical features demonstrating and predicting probability of survival. **(B)** Expression and prognostic analysis of model genes. **(C)** Heatmap of the risk score distribution, patient survival. and model gene expression for the TCGA cohorts. **(D)** The Univariate Cox regression analysis of subtype clinical characteristics and subtyping with respect to overall survival. **(E)** The multivariate Cox regression analysis of subtype clinical characteristics and subtyping with respect to overall survival. **(F)** Distribution of clinical features in high and low-risk subgroups. **(G)** Heatmap depicting the distribution of risk subgroups in Hallmark signalling sets. **(H)** PROGENy probing altered tumor signalling pathways in risk subgroups (Wilcox. test). **(I)** Expression of EMT-associated transcripts between risk subgroups. *. p < 0.05. **, p < 0.01. ****, p < 0.001.

In addition, the Nomogram showed good predictive value of T Stage, N Stage and Risk Score for TCGA cohort samples (Figure 3A), Calibration curve showed good predictive value of the model at 1, 3 and 5 years (Supplementary Figure S1E), Decision Curve Analysis The results show that Riskscore has good application value (Supplementary Figure S1F). According to these results, we hypothesized that Riskscore is a reliable predictor and is associated with poor prognosis.

Furthermore, we analysed 3 model genes as well. PRC1, TNS4, and CDKN3, which were used to construct the model, had significantly higher mRNA expression in lung adenocarcinoma samples than normal samples in the TCGA cohort (Figure 3B). Protein expression levels in tissues were analysed using the HPA data frame, and protein expression levels of PRC1 were higher in LUAD tissues than in normal tissues (Supplementary Figure S2A). In addition, three genes showed a significant relationship with prognosis in LUAD samples in survival analysis (Figure 3B) and had a better diagnostic effect in the results of the Receiver Operating Characteristic Curve analysis (Supplementary Figure S2B).

By evaluating the model as a whole and evaluating the genes of the model separately, the results of the analyses show that the model has good predictive efficacy. Therefore, we will analyze the differences in clinical characteristics and molecular processes between the high- and low-risk groups.

### Unique clinical features and molecular processes in subgroups

We first summarised the risk scores, survival, and model gene expression in the two subgroups (Figure 3C). We found that patients in the low-risk subgroup survived significantly better than those in the high-risk subgroup, while model gene expression was significantly lower than that in the high-risk subgroup. We also performed Univariate Cox regression analysis (Figure 3D) and Multivariate Cox regression analysis (Figure 3E) for clinical characteristics, and risk scores. The results showed that Riskscore was associated with survival outcomes in lung adenocarcinoma patients both as an independent factor as well as in the case of adjustment for clinical factors, and was present as a risk factor. The high and low-risk subgroups were statistically significant in the distribution of clinical characteristics (Figure 3F). Notably, we found that Riskscore was statistically significantly associated with Stage distribution, M Stage, and T Stage distribution of LUAD samples, i.e., increased Riskscore was often accompanied by worse stage occurrence as well as tumor metastasis in LUAD samples (Supplementary Figure S1G).

To further explain the differences in clinical characteristics between high- and low-risk subgroups, we attempted to explore molecular pathway alterations between subgroups. We performed gene set variation analysis (GSVA) using the Hallmark gene set. The results showed that the pathways associated with cancer cell proliferation and metastasis were significantly enriched as the score increased (Figure 3G), such as GLYCOLYSIS, DNA_REPAIR, MYC_TARGETS_V2, UNFOLDED_PROTEIN_RESPONSE, MTORC1_SIGNALING, G2M_CHECKPOINT, E2F_TARGETS, MITOTIC_SPINDLE, PI3K_AKT_MTOR_SIGNALING, HYPOXIA. In particular, LPS can lead to an increase in ROS(Beutler, 2004; Pchejetski et al., 2011), which can mediate MMP-3-induced EMT and genomic instability (Radisky et al., 2005); Unfolded Protein Response (UPR), as one of the most important adaptive systems of tumor cells, can adapt to external stimuli by integrating multiple signaling pathways to promote tumor cell survival, and has been shown to be associated with EMT (Shen et al., 2015). PROGENy enrichment analysis further validated the molecular pathway differences between the two subgroups, and showed that the high-scoring subgroup received significant enrichment in pathways related to tumor progression and metastasis, including EGFR, Hypoxia, JAK-STAT, MAPK, NFkB, PI3K, TNFa, Trail, VEGF and WNT pathways (Figure 3H). On the basis of these results, we speculate that Riskscore has some relationship with EMT, and that this progression of epithelial-mesenchymal transition may occur through a signalling cascade of pathways such as hypoxia, MAPK, PI3K, VEGF, and WNT. In addition, we also collected transcripts associated with the progression of EMT. We found that the expression was significantly higher in the high-risk subgroup than in the low-risk subgroup (Figure 3I).

**FIGURE 4:**
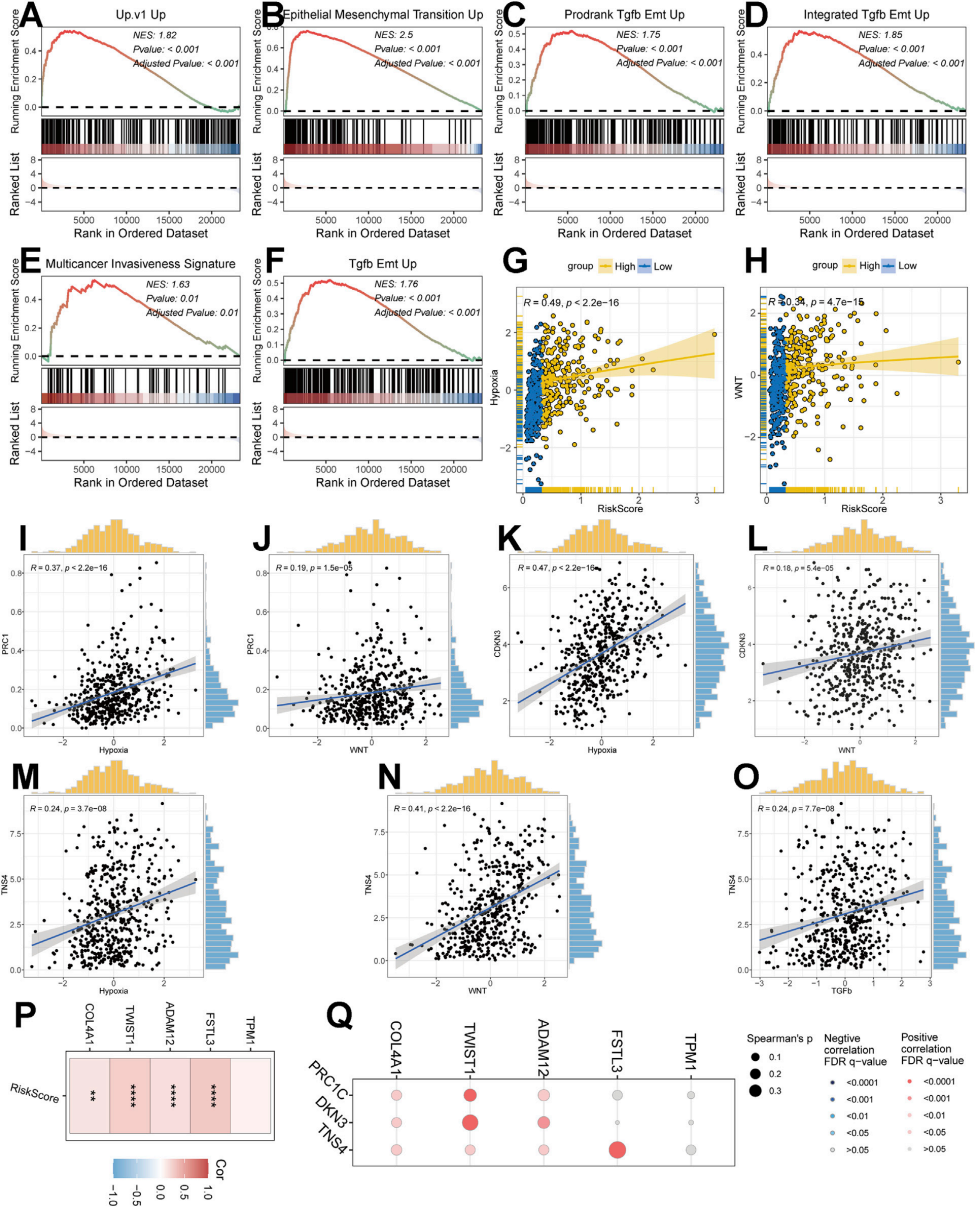
Correlation of risk modeling with EMT progression. **(A–F)** GSEA discriminating pathway enrichment in high and low-risk subgroups. **(G,H)** Correlation of Riskscore with the EMT pathway (Hypoxia, WNT pathway). **(I–O)** Correlation analysis of model genes with the EMT pathway. **(P)** Correlation of Riskscore with EMT-related transcripts. **(Q)** Correlation of model genes with EMT-related transcripts.

GSEA is used to study sets of marker genes that are differentially enriched between high and low-risk subgroups. To further validate that the high-risk subgroup has high EMT status, we collected six EMT-related gene sets (Up.c1_Up, Epithelial_Mesenchymal_Transition_Up, Prodrank_Tgfb_Emt_Up, Integrated Tgfb_Emt_uP, Multicancer_Invasiveness_Signature, Tgfb_Emt_Up) and performed GSEA, which showed that highly expressed genes in the high-risk subgroups were all significantly enriched in the EMT gene set (Figures 4A–F). This result likewise confirms our conjecture that risk score has a relationship with EMT progression.

### Correlation between Riskscore and EMT

To further confirm the potential dependence of Riskscore on EMT progression, we analysed multiple indications. Firstly, we found that Riskscore had a good dependence with Hypoxia pathway (R = 0.49, p < 2.2e-16) and WNT pathway (R = 0.34, p = 4.7e-15) (Figures 4G,H). The analysis of model genes PRC1, CDKN3 and TNS4 showed that PRC1 had good correlation with WNT pathway and Hypoxia pathway (Figures 4I,J), CDKN3 had good correlation with WNT pathway and Hypoxia pathway (Figures 4K,L), TNS4 had good correlation with WNT pathway, Hypoxia pathway, TGFb pathway existed better correlation (Figures 4M–O). In addition, we found that the expression of Riskscore, model genes and EMT-related transcripts were all well correlated (Figures 4P,Q).

Based on the above analysis of molecular alterations, we found that the risk model was closely associated with the progression of EMT and correlated with pathways such as MAKP, PI3K, and WNT.

### Unique TME infiltration patterns in subgroups

**FIGURE 5:**
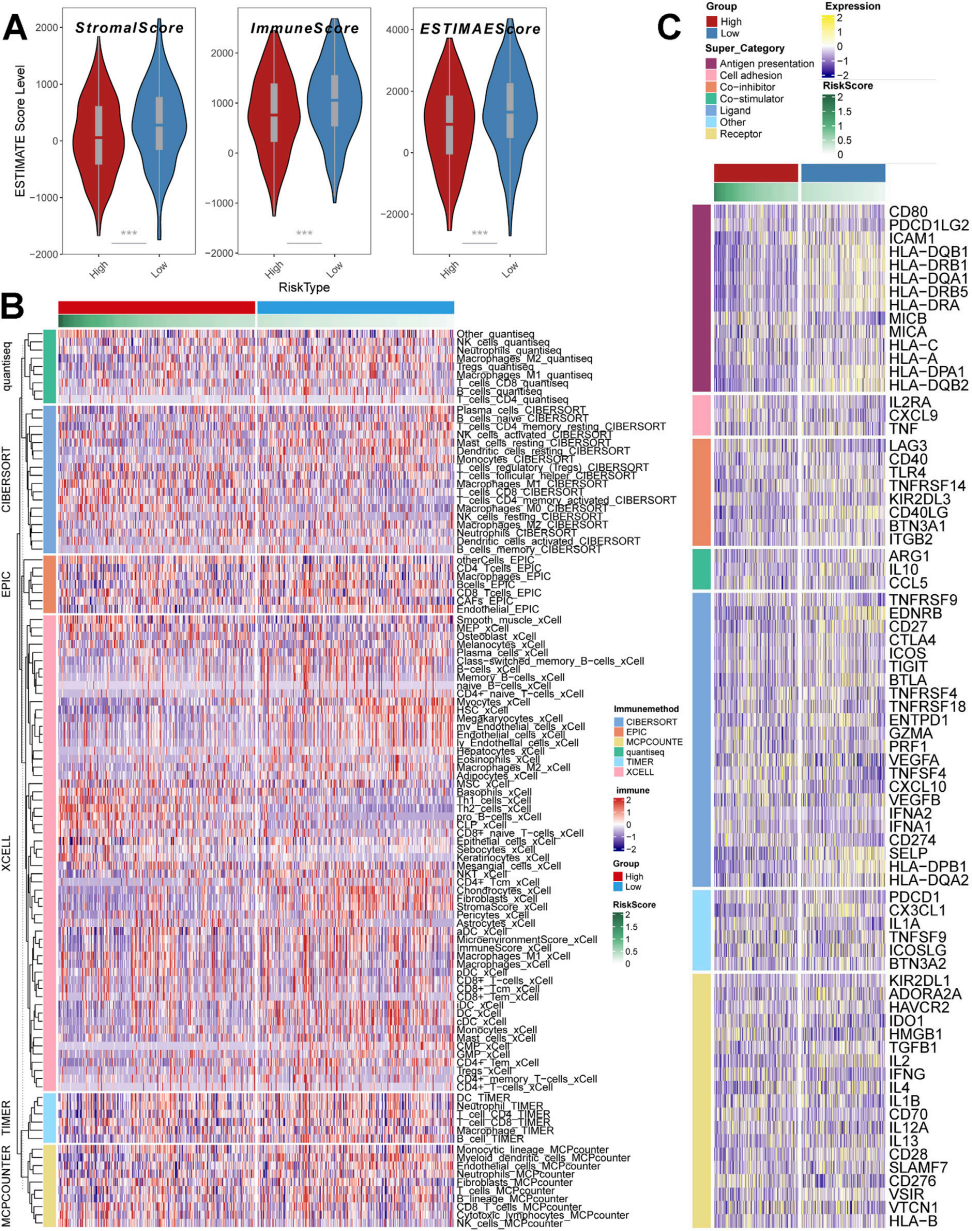
Depiction of unique TME infiltration patterns between subgroups. **(A)** ESTIMATE scores of risk subgroups (Wilcox. test) **(B)** Heatmap demonstrating the variability of cellular infiltration levels between risk subgroups **(C)** Heatmap demonstrating the expression of immune-related transcripts between risk subgroups. *, p < 0.05. **, p < 0.01. ***, p < 0.001.

Further analyses are still needed for changes in EMT progression in risk models. The tumor microenvironment (TME) occupies an important role in promoting lung carcinogenesis (Jin and Jin, 2020), and we analysed cellular infiltration in two molecular subgroups. Firstly, we quantified the overall immune infiltration level of the two risk subgroups using the ESTIMATE algorithm (Figure 5A) and found that ImmuneScore and StromalScore had a higher level in the low-risk subgroups, which suggests that there is a higher infiltration of immune cells in the tumor microenvironment of our low-risk subgroups. On this basis, we used the IOBR algorithm to explore the variability of cellular infiltration patterns in the subgroups and found that most cell categories were significantly different in the two subgroups (Figure 5B). Overall, the high-risk subgroup was characterised by infiltration of smooth muscle cells and epithelial cells; the low-risk subgroup was characterised by infiltration of CD8^+^ T cells and B cells.

Additionally, we acquired transcripts associated with Antigen presentation, Cell adhesion, Co-inhibitor, Bo-stimulator, Ligand, Receptor, and other types (Figure 5C) and explored them in two risk subgroups. The results showed differences in transcript expression between the two subgroups, and in particular, we found that Antigen presentation-associated transcripts were significantly higher in the low-scoring subgroup than in the high-scoring subgroup. This result suggests that the low-scoring subgroup may respond to the immune response through antigen presentation, resulting in a significantly better prognosis in the low-scoring subgroup than in the high-scoring subgroup.

### Mutational characterisation of subgroups and value in the prediction of response to immunotherapy

**FIGURE 6:**
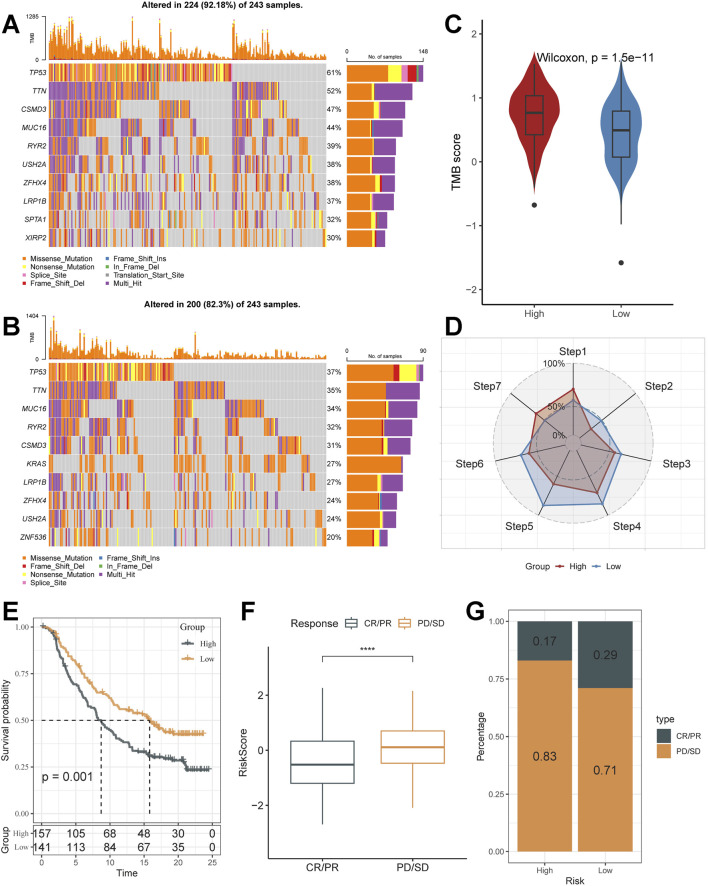
Unique mutational landscapes of subgroups and the value of Riskscore in immunotherapy. **(A,B)** Unique gene mutation landscapes of high and low-risk subgroups. **(C)** TMB Score display for high and low-risk subgroups (Wilcox. test). **(D)** Anti-cancer immune activity of subgroups in the cancer-immunity cycle. **(E)** Kaplan-Meier curves for the high and low-risk score groups in the IMvigor210 cohort. **(F)** Difference in morbidity risk scores between the PD/SD and CR/PR groups in the IMvigor210 cohort (Wilcox. test). **(G)** Distribution of anti-PD-1 treatment responses in different risk subgroups. *, p < 0.05. **, p < 0.01. ***, p < 0.001. ****, p < 0.0001.

Single nucleotide polymorphism (SNP) profiles differed in the top 10 genes of the two scoring subgroups (Figures 6A,B). Mutation rates vary widely, even in genes shared between them. For example, TP53, a well-recognised oncogene, was found to be mutated in 61% of the high-scoring subgroup and only 37% of the low-scoring subgroup. Previous studies have shown that TP53 mutations are the most enriched mutations in the invasive phase of lung adenocarcinoma and that TP53 is a key mediator of lung cancer invasion (Chen et al., 2019). This result suggests that there may be a stronger invasiveness in the high-scoring subgroup.

Cancer treatment has been revolutionised by the advent of cancer immunotherapy, the success of which relies heavily on the development and activation of immune cells in the system (Daud et al., 2016). Systematic tracking of tumor immune phenotypes is essential for understanding the mechanisms of tumor immunity and improving the clinical efficacy of immunotherapy. Tumor tissue is heterogeneous, with varying tumor mutation burden (TMB) (Figure 6C), and presents a correlation with durable clinical response to anti–PD-1/PD-L1 immunotherapy. Before performing the immunotherapy response prediction analysis, we first analysed the cancer-immunity cycle between subgroups (Figure 6D). The results showed that the high-scoring subgroup had significantly higher levels than the low-risk subgroup in Step 1: Release of cancer cell antigens, Step 7: Killing of cancer cells; while the low-risk subgroup had significantly higher levels than the low-risk subgroup in Step 2: Cancer antigen presentation, Step 3: Priming and activation, step 4: Trafficking of immune cells to tumors, step 5: Infiltration of immune cells into tumors, and step 6: Recognition of cancer cells by T cells were expressed at a higher level of expression. This finding matches the results of the mutation analysis that high TMB levels in high-risk subgroups lead to more antigen production, which in turn promotes steps one and seven. The prognosis of the low-risk subgroup in the IMvigor210 cohort was better (Figure 6E), and its proportion of Partial response (PR), Complete response (CR) after treatment was higher than that of the high-risk subgroup (Figures 6F,G). It was also validated in the GSE78220 cohort (Supplementary Figure S2C–E). The low-risk subgroup had better tumor immunotherapy benefit, which may be associated with its active status in multiple steps of tumor immunity.

In the above analysis, the process of increased Riskscore scores was accompanied by enrichment of cancer cell proliferation metastasis-related pathways, lower levels of immune cell infiltration, and higher TMB; the high-risk subgroup demonstrated significant signal generation of epithelial-mesenchymal transition and mutational signals associated with invasion (e.g., TP53 mutations), results that may contribute to a low-benefit effect in tumor immunotherapy. We also found a strong correlation between Riskscore and EMT-related pathways. Consequently, we speculated that Riskscore is associated with EMT and that as the score increases, it tends to lead to tumors producing an EMT phenotype as well as greater aggressiveness and low benefit in tumor immunotherapy.

### Identification of the application value of EMT-risk signature

**FIGURE 7:**
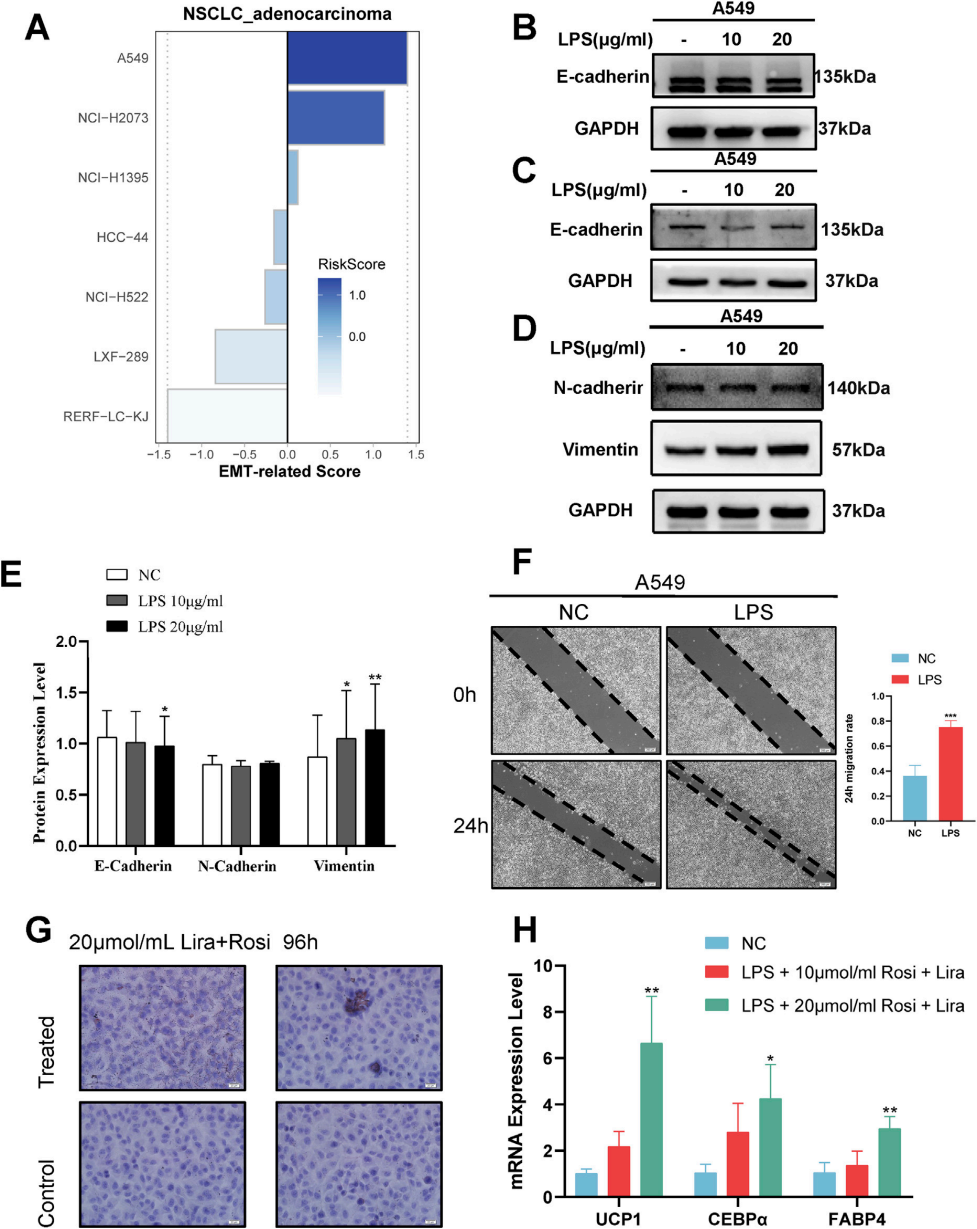
Identification and validation of EMT-related Signature in in vitro experiments. **(A)** Lung adenocarcinoma cell line EMT-related Signature visualised. **(B)** Western blot results of epithelial markers at 48 h of LPS induction. **(C)** Western blot results of epithelial markers at 72 h of LPS induction. **(D)** Western blot results of mesenchymal markers at 72 h of LPS induction. **(E)** Densitometric images of markers 72 h after LPS induction. **(F)** Representative images and statistical analysis of wound healing test after induction (t.test). **(G)** Oil red O staining assay demonstrating the formation of lipid droplets in cancer cells after the lipogenic induction regimen. **(H)** RT-PCR detection of adipocyte marker expression after the lipogenic induction regimen (One-way ANOVA)*, p < 0.05. **, p < 0.01. ***, p < 0.001.

Basing on the above analyses, we defined Riskscore as a score that correlates with the EMT status of cancer cells, i.e., EMT-risk signature. In order to identify its application value *in vitro* LPS induction experiments, we further collected the expression profiles of lung adenocarcinoma cell lines from the Cancer Cell Line Encyclopedia (CCLE) project (Ghandi et al., 2019) and assessed specific EMT-risk signature based on the expression profiles of each cell line and presented some of the cell lines’ EMT-risk signatures were demonstrated (Figure 7A). We selected the human lung adenocarcinoma cell line A549 for subsequent *in vitro* experimental validation.

A549 cells were induced by exposing them to different concentrations of LPS, and Western blot results at 48 h showed that the epithelial marker E-cadherin was not significantly down-regulated (Figure 7B). At 72 h, Western blot showed a decrease in the expression of epithelial marker E-cadherin and an increase in the expression of mesenchymal marker Vimentin (Figures 7C–E); meanwhile, the wound healing assay showed a significant increase in the migration rate of A549 cells after LPS induction (Figure 7F). This indicates that the cancer cells undergo EMT progression and have high invasiveness after LPS induction.

### Potential therapeutic possibilities for highly aggressive EMT cancer cells

**FIGURE 8:**
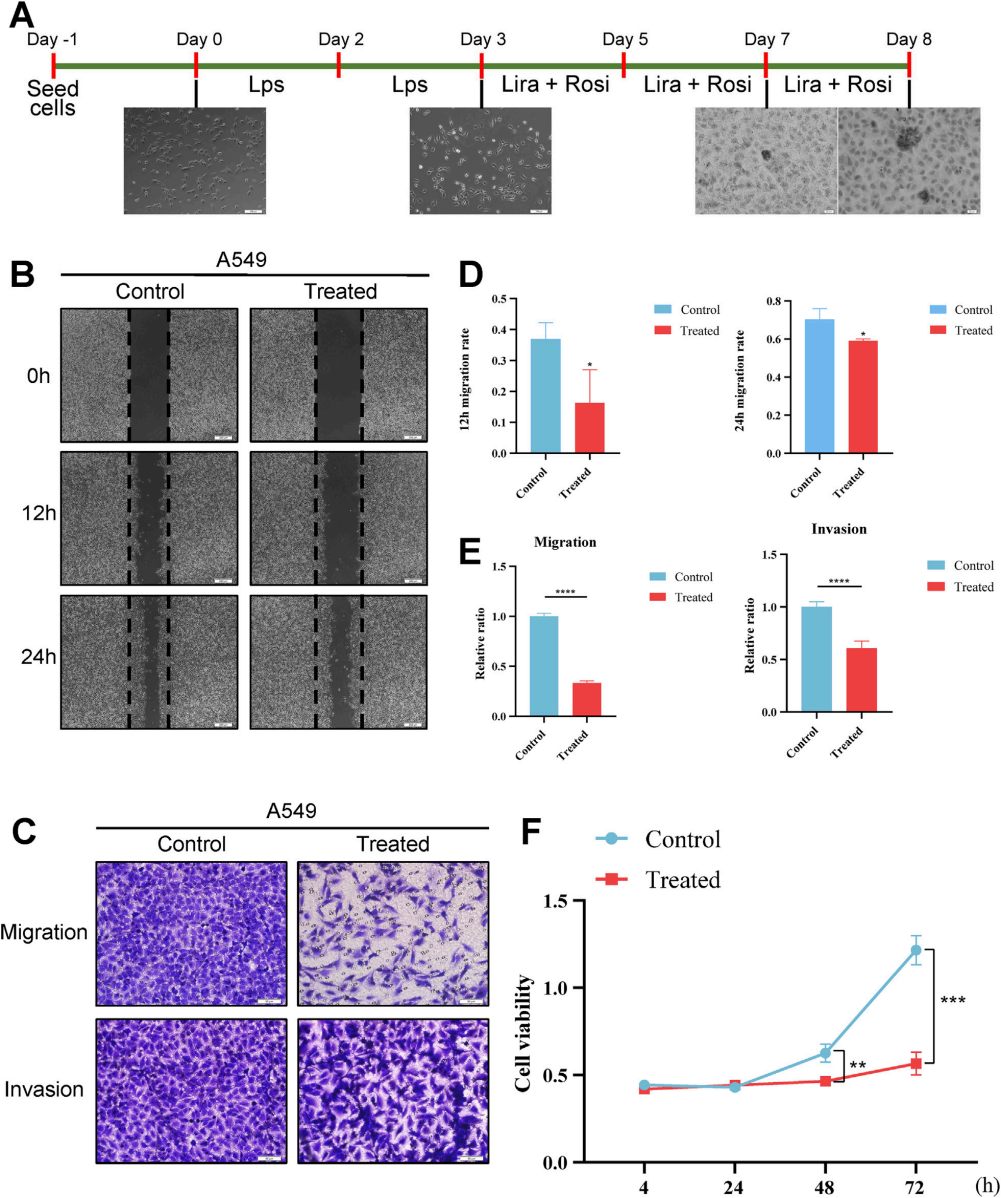
Effect of lipidogenic induction programme on cellular phenotype. **(A)** Schematic diagram of the fat-forming transdifferentiation induction protocol. **(B)** Representative images of the post-treatment wound healing assay. **(C)** Representative images of the Transwell assay. **(D)** Statistical analysis of the post-treatment wound healing assay (t.test). **(E)** Statistical analysis of the results of the Transwell assay (t.test). **(F)** CCK-8 assay for detecting the effects of fat-forming transdifferentiation induction on the proliferation of cells effects (t.test). *, p < 0.05. **, p < 0.01. ***, p < 0.001. ****, p < 0.0001.

Considering that EMT occurs during tumor development, then the use of plasticity in the EMT state to trans-differentiate highly invasive cancer cells into less invasive cells becomes a new idea for cancer treatment. Following the success of LPS in inducing EMT-like alterations in A549 cells, we identified a possible novel therapeutic modality, a combined induction regimen of Rosiglitazone (Rosi) and Liraglutide (Lira). After 96 h of combined induction using Rosi and Lira, the results of the oil red O fat staining assay showed increased lipogenesis in cancer cells (Figure 7G). Meanwhile, RT-qPCR results showed upregulated and statistically significant expression of UCP1, C/EBPα, and FABP4 mRNA (Figure 7H). At different stages of induction, the morphology of cancer cells also changed accordingly (Figure 8A), e.g., the cells changed from normal paving-stone shape to epithelial-mesenchymal spindle and polygonal shape at the stage of EMT. At the same time, obvious lipid droplets were produced in the cancer cells after the *in vitro* lipogenic induction protocol. We define this *in vitro* combined induction protocol as a “lipogenic trans-differentiation induction protocol”.

We examined the effect of a lipogenic trans-differentiation induction protocol on the phenotype of cancer cells. Scratch assay showed that A549 cells induced by lipid differentiation *in vitro* showed a significant decrease in scratch healing ability compared to the control group not induced by the lipid differentiation protocol (Figures 8B,D). CCK-8 assay showed that the OD value of the A549 cells in the lipid differentiation induction group was significantly lower, and the cellular activity was reduced compared to the control cells not induced by the lipid differentiation protocol (Figures 8C,E). Transwell assay showed that the number of cells penetrating through the chambers and stromal gel was statistically significantly lower in A549 cells treated with the induction protocol compared to the control group (Figure 8F).

## Discussion

The prognosis of patients is worsened by concomitant lung infections. The presence of LPS, a constituent of the cell membrane of Gram-negative bacteria, has been linked to an upsurge in inflammatory cytokines and ROS production, and has been linked to tumor metastasis, invasion, and EMT transformation (Park et al., 2017; Xia et al., 2021). The mechanism of the role of LPS in malignant progression of lung adenocarcinomas and possible therapeutic strategies still need to be explored. Therefore, we explored the LPS-induced signature genes and constructed the LPS-induced lung adenocarcinoma risk model at the transcriptome level for the first time, and performed systematic bioinformatics analysis. By characterizing subgroups with unique clinical features, pathway changes, cellular infiltration and mutational features, we found a strong correlation between the risk model and EMT progression. We selected cell lines with high risk scores for LPS induction experiments and successfully constructed a cancer cell model for EMT progression and utilized high plasticity for transdifferentiation therapy. This may provide new insights into more effective treatment strategies for patients with LPS-infected lung adenocarcinoma and metastatic lung adenocarcinoma.

We obtained LPS-induced signature genes by weighted correlation network analysis and differential expression analysis. Based on the signature genes, we designed a novel risk scoring scheme associated with LPS induction and delineated two distinct subgroups that showed significant differences. All three model genes in the scoring scheme are differentially expressed in lung adenocarcinoma and are associated with poor prognosis in lung adenocarcinoma. Furthermore, they all act as reliable indicators for diagnosis. Simultaneously, PRC1 was found to be linked to TGF-β-induced epithelial-mesenchymal transition-related lung and pancreatic cancer cell lines (Wanna-Udom et al., 2021); CDKN3 has a role in regulating proliferation, invasion, and EMT transition in nasopharyngeal and lung cancers (Wang et al., 2011; Xing et al., 2012; Gao et al., 2019); TNS4 was reported to be associated with regulation of cell adhesion, motility, invasion and EMT in colorectal cancer (Raposo et al., 2020).

We performed a systematic bioinformatics analysis of the risk model. First, in terms of clinical characteristics, the high-risk subgroups had a worse prognosis and were characterized by worse clinical presentations. Further, we investigated molecular pathway changes between subgroups to reveal the reasons behind the significant differences in clinical characteristics between subgroups. Pathways associated with cancer and tumor metastasis were significantly enriched in the high-risk subgroup, including the PI3K pathway, EGFR pathway, hypoxia pathway, VEGF pathway, and WNT pathway, whereas immune-related pathways were more pronounced in the low-risk subgroup, including the IL2-STAT pathway. Considering that the model genes were derived from LPS, we hypothesized that the high-risk subgroup with highly tumor invasive and metastasis-related features was associated with metastatic and highly invasive tumors due to EMT. The expression profiles of EMT-related transcriptional markers and the enrichment of EMT-related pathways confirmed our hypothesis. In addition, we found strong correlations between Riskscore, model genes, and EMT-related pathways, including hypoxia and WNT pathways, among others. In addition, at the level of TME infiltration, we found that the low-risk subgroup had a higher immune infiltration status, such as CD8^+^ T cells and B cells; whereas the high-risk subgroup had more stromal cell aggregates, such as smooth muscle cells and epithelial cells. The low-risk subgroup had higher expression levels of antigen presentation-related transcripts. This implies that the low-risk subgroup exhibits a corresponding immune response by presenting antigens, and similar results were observed in the cancer immune cycle. The two subtypes also differed significantly in their mutational profiles, with the particular high-scoring subgroup having significant mutations in the oncogene TP53, aligning with prior research indicating a correlation between TP53 mutations and invasive lung adenocarcinomas, and that elevated TP53 mutations typically signal the development of invasion (Zhang et al., 2019). With these findings in mind, we conclude that the LPS-induced risk model is significantly associated with EMT progression. As the score rises, the EMT phenotype and aggressiveness of the tumor is made more pronounced and the efficacy of tumor immunotherapy is diminished. In addition, the same results were obtained when applying our model to the IMvigor210 and GSE78220 cohorts, i.e., low-scoring patients achieved better clinical outcomes after anti-PD-L1 and anti-PD-1 treatments, affirming the predictive validity of our predictions. We selected lung adenocarcinoma cell lines with high scores for *in vitro* experimental validation to further verify the accuracy of the scores by inducing EMT progression of cancer cells using LPS.

During metastasis, lung adenocarcinoma cells become more invasive potential through dedifferentiation (Lawson et al., 2015), with EMT being the key to tumor cell invasion and metastasis. The use of high plasticity in the EMT state to trans-differentiate highly invasive cancer cells into less invasive cells is a new idea for the treatment of cancer. Ishay-Ronen D and colleagues conducted the research. The combination of a MEK inhibitor and the antidiabetic drug rosiglitazone was used to induce the transformation of invasive breast cancer cells in the EMT state into postmitotic adipocytes, thus preventing primary tumor invasion and metastasis formation (Ishay-Ronen et al., 2019). This pioneering research has been equally enlightening. Peroxisome proliferators-activated receptors (PPARγ) are nuclear hormone receptors activated by thiazolidinediones antidiabetic drugs and are implicated in adipogenesis, lipid metabolism and insulin sensitivity (Tontonoz and Spiegelman, 2008; Wang et al., 2018; Hernandez-Quiles et al., 2021). PPARγ seems to function as both a direct controller of numerous adipose-specific genes and a “master” controller that initiates the adipogenic program (Martinez Calejman et al., 2020). Rosiglitazone, a PPARγ agonist, has been demonstrated to trigger cellular redifferentiation in various malignancies (Prost et al., 2015), whereas research has shown that liraglutide stimulates AMKP/PGC1α to facilitate adipocytogenesis (Zhou et al., 2019). In this study, the combination of rosiglitazone and liraglutide induced lipogenic trans-differentiation, resulting in increased lipid droplet production in cancer cells, as well as up-regulation of the expression of the mature adipose marker FABP4, the adipocyte-specific markers CEBPα, and UCP1 in RT-qPCR. This result suggests that such cancer cells may be biased towards a “cancer-adipocyte”. Following the successful implementation of the fat-forming trans-differentiation induction protocol, we investigated the modified characteristics of cancer cells, and the CCK8 findings indicated that lung adenocarcinoma cells undergoing the EMT process exhibited a diminished ability to proliferate when combined with the fat-inducing regimen, thereby implying the potential for cancer cells to undergo trans-differentiation. Subsequently, we discovered that the capacity of cancer cells to migrate and invade was diminished by cell scratch assay and Transwell assay. Research conducted *in vitro* experiments has indicated that LPS-induced EMT cancer cells with a high level of invasiveness may be able to diminish tumor spread and cancer growth by using a lipogenic trans-differentiation technique to treat malignant growths.

Inevitably, this study needs to explain some of its limitations. Firstly, the study cohorts were drawn from public databases, which leads to an inherent case selection bias that may affect the results, and more convincing prospective studies are needed to confirm our findings. Second, due to the limited sample size, large-scale cohort studies are essential to assess the value of the model. Meanwhile, based on the bioinformatics identification of the EMT-related Signature as well as its model genes, they both require further experiments to investigate their underlying biological mechanisms. Lastly, there are still shortcomings in the study of rosiglitazone and liraglutide lipogenic trans-differentiation induction protocol in lung adenocarcinoma. For example, due to the deviation of the concentration of the drug itself may lead to unsatisfactory trans-differentiation results, and the potential mechanism of the lipid-forming trans-differentiation regimen in inhibiting the malignant invasion process of lung adenocarcinoma cells still needs to be further explored; meanwhile, the present study has achieved a certain degree of effect *in vitro* experiments, and it's *in vivo* experiments still need to be further explored.

Taken together, we identified a class of hub genes related to LPS-induced lung cancer at the bioinformatics level. We constructed and validated an EMT-related Signature based on the hub genes for the first time. The scoring subgroups showed significant differences in clinical features, TME patterns, pathway change, mutations atlas, and immunotherapy response. It was also validated *in vitro* experiments and found a potentially possible therapeutic option that exploits the high plasticity of cancer cells during EMT. And it was validated *in vitro* experiments. We hope that the findings will be beneficial in advancing research into the progression of EMT in LPS-induced cancers, as well as in offering fresh perspectives on trans-differentiation therapy for invasive LUAD cells.

## Data Availability

The datasets presented in this study can be found in online repositories. The names of the repository/repositories and accession number(s) can be found in the article/Supplementary Material.
