# Supplementary material for: Comprehensive bioinformatics analysis of malignant transformation and potential therapeutic possibility of lung adenocarcinoma after lipopolysaccharide induction
Source: Front Genet. 2025 Jul 8;16:1556366. doi: 10.3389/fgene.2025.1556366 (PMC12279497; doi:10.3389/fgene.2025.1556366)
Supplement: Supplementary file 2 [file Supplementaryfile1.docx]

**
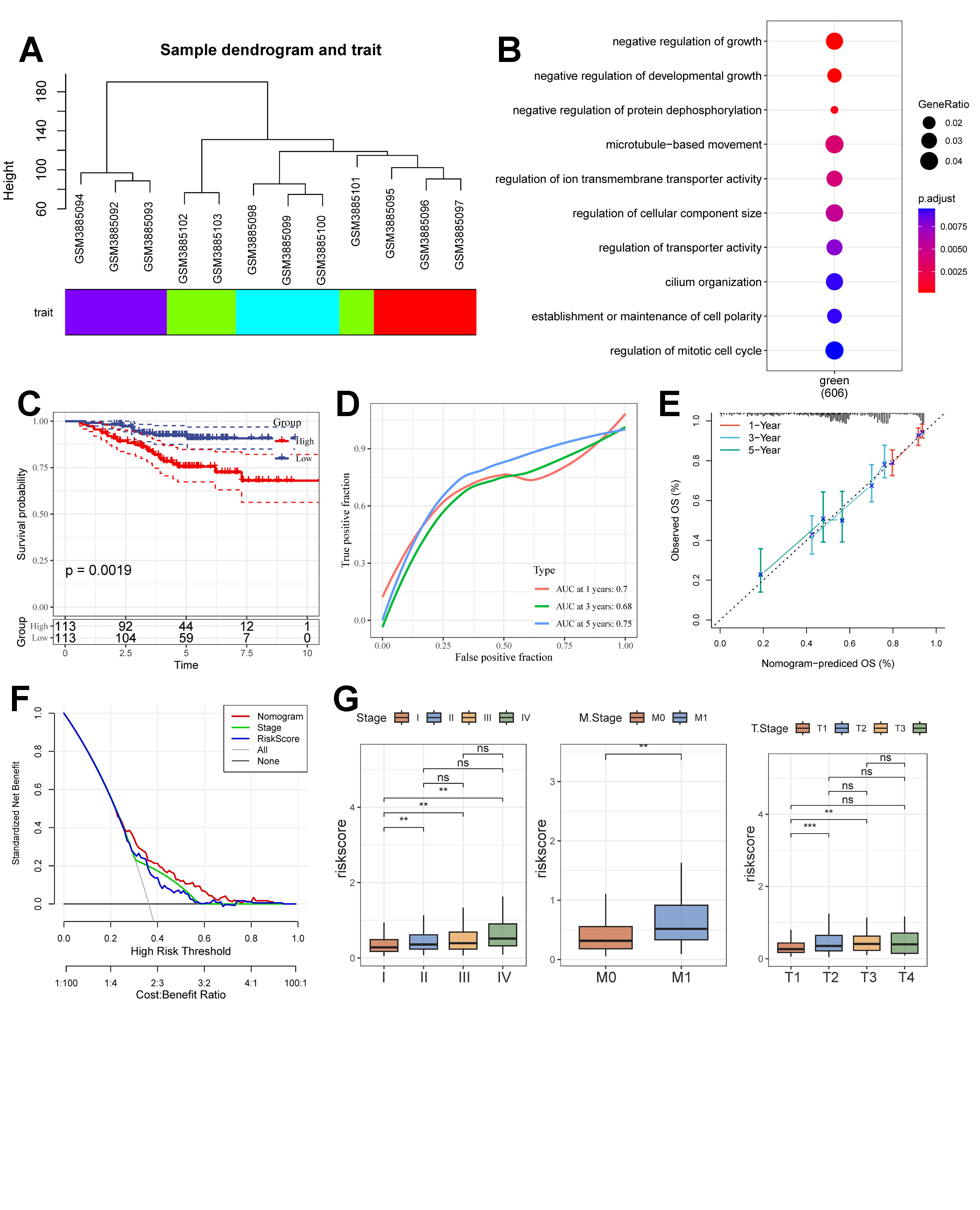
Supplementary Figure.1 Analysis related to risk modeling.**

**(A) Distribution of samples in WGCNA (B) Results of GO enrichment analysis of Green module genes （C）Kaplan-Meier curves for the high and low-risk score groups in the GSE31210 cohort. （D）the Receiver Operating Characteristic Curve analysis for GSE31210 cohorts. (E) Nomogram calibration curves for 1, 3, and 5 years. (F) Decision Curve Analysis (DCA) demonstrating net benefit by applying nomogram and other clinical features.** **(G) Demonstration of clinical features associated with tumor metastasis in high and low-risk subgroups (Wilcox. test). *, p < 0.05. **, p < 0.01. ***, p < 0.001.**

**
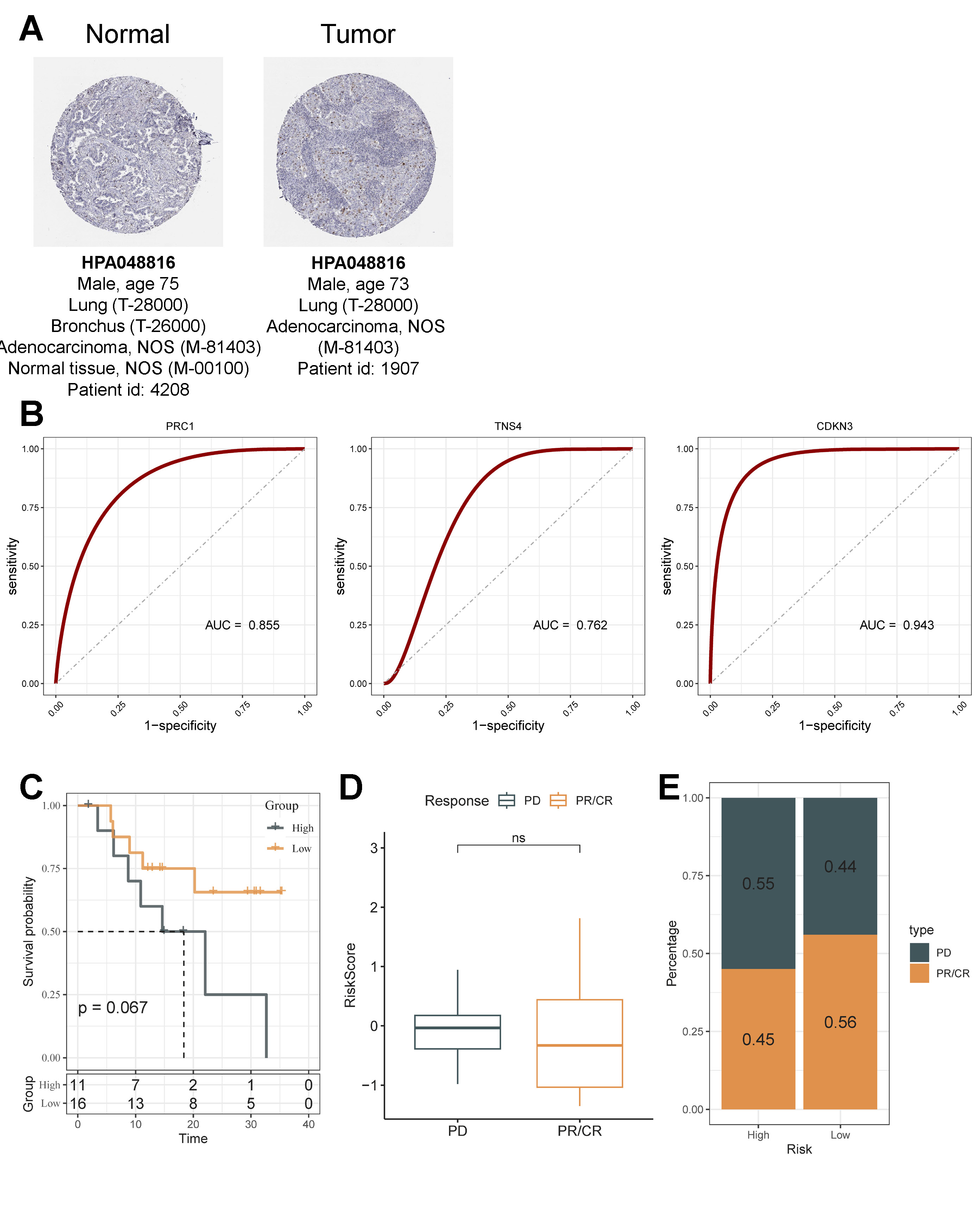
**

**Supplementary Figure.2 Model gene analysis and immunotherapy cohort analysis.**

**(A) Verification of model gene expression in LUAD and normal lung tissue using the HPA database. (B) The Receiver Operating Characteristic Curve of PRC1, TNS4, and CDKN3 in LUAD. (C) Kaplan-Meier curves for the high and low-risk score groups in the GSE78220 cohort. (D) Difference in morbidity risk scores between the PD/SD and CR/PR groups in the GSE78220 cohort (Wilcox. test) (E) Distribution of anti-PD-1 treatment responses in different risk subgroups.**
